# Determinants of vascular function in patients with type 2 diabetes

**DOI:** 10.1186/1475-2840-11-127

**Published:** 2012-10-12

**Authors:** Katerina K Naka, Katerina Papathanassiou, Aris Bechlioulis, Nikolaos Kazakos, Konstantinos Pappas, Stelios Tigas, Dimitrios Makriyiannis, Agathocles Tsatsoulis, Lampros K Michalis

**Affiliations:** 1 Michaelidion Cardiac Center, University of Ioannina, Ioannina, Greece; 2 Department of Cardiology, University of Ioannina, Ioannina, Greece; 3 Department of Endocrinology, University of Ioannina, Ioannina, Greece; 4 Department of Endocrinology, Hatzikosta General Hospital, Ioannina, Greece; 5 Department of Cardiology and Michaelidion Cardiac Centre, University of Ioannina, Ioannina, Greece

**Keywords:** Type 2 diabetes mellitus, Pulse wave velocity, Flow-mediated dilation, Nitrate-mediated dilation, Atherosclerosis

## Abstract

**Background:**

Type 2 diabetes mellitus (T2DM) is independently associated with an increased risk for cardiovascular diseases that is primarily due to the early development of advanced atherosclerotic vascular changes. The aim of our study was to investigate the predictors of vascular dysfunction in T2DM patients.

**Methods:**

We studied 165 T2DM patients without known macrovascular or microvascular disease. Standard demographic (age, gender, cardiovascular risk factors, medications), clinical (body mass index, blood pressure) and laboratory (glucose, glycated hemoglobin, lipids, renal function) parameters were included in analyses. Brachial artery flow-mediated dilation (FMD), nitrate mediated dilation (NMD) and Carotid-Femoral Pulse Wave Velocity (PWV) were measured.

**Results:**

Median age was 66 years and duration since T2DM diagnosis was 10 years, 70% were females and 79% hypertensives, while only 10% had a glycated hemoglobin <7%. FMD was positively associated with NMD (r 0.391, P < 0.001), while PWV was inversely associated with FMD (r -0.218, P = 0.014) and NMD (r -0.309, P < 0.001). Time since diagnosis of diabetes was the single independent predictor of FMD (β -0.40, P = 0.003). Increased age and fasting glucose and the presence of hypertension were independent predictors of decreased NMD (P < 0.001). Increased age and systolic blood pressure were independently associated with increased PWV (P < 0.001).

**Conclusions:**

In T2DM patients, impairment of endothelium-dependent vasodilation was independently associated only with longer diabetes duration while no association with other established risk factors was found. Vascular smooth muscle dysfunction and increased arterial stiffness were more prominent in older T2DM patients with hypertension. Worse glycemic control was associated with impaired vascular smooth muscle function.

## Background

Type 2 diabetes mellitus (T2DM) is independently associated with an increased risk for cardiovascular diseases (CVD) [1] that is primarily due to the early development of advanced atherosclerotic vascular changes [2]. Vascular endothelial and smooth muscle cell dysfunction as well as large arterial stiffness are considered to be markers of subclinical atherosclerosis with a significant prognostic role in high risk populations [3-10]. Endothelial dysfunction, as assessed by decreased endothelium-dependent flow-mediated dilation (FMD) of the brachial artery [11-19], and increased arterial stiffness, assessed by aortic pulse wave velocity (PWV) [7,20-22], have been previously demonstrated in patients with T2DM compared to controls. Whether vascular smooth muscle cell function, assessed by nitrate mediated dilation (NMD) of the brachial artery [11-13,15,16,18,23] is also affected in T2DM patients has been questioned in previous studies.

The mechanisms underlying the association of T2DM with vascular dysfunction are considered to be complex. Classical cardiovascular risk factors (hypertension, dyslipidemia and smoking) may play a role, while diabetes-related parameters such as hyperglycemia, insulin resistance and obesity, and other associated emerging risk factors such as inflammation, may also contribute to the impairment of arterial function indices in T2DM. The relative importance of these risk factors in the induction of vascular dysfunction in T2DM patients has not been well studied previously.

The aim of our study was to investigate the predictors of vascular function, as assessed by brachial artery FMD and NMD and carotid-femoral PWV in T2DM patients without known macrovascular or microvascular disease.

## Methods

### Subjects

A total of 165 patients with previously diagnosed type 2 diabetes mellitus were consecutively recruited from the Endocrinology outpatient clinics of the University Hospital and Hatzikosta General Hospital of Ioannina, Ioannina, Greece from 2007 to 2009. Diagnosis of type 2 diabetes mellitus was defined according to the American Diabetes Association and the European Association for the Study of Diabetes [24]. Eligible subjects were patients 40 – 80 years old, under stable treatment with oral antidiabetic medications (metformin, sulfonylureas) or/and insulin for at least 6 months prior to study enrollment. No patient was on thiazolidinedione therapy. The duration of diabetes was defined as time since T2DM diagnosis and was confirmed by the patients’ clinical records. Patients reporting a history of macrovascular disease (coronary artery, cerebrovascular, or peripheral vascular disease), microvascular disease (diabetic retinopathy, symptomatic neuropathy, proteinuria), chronic heart failure, liver disease (or abnormal liver enzymes at study entry), anemia, thyroid dysfunction or other endocrine diseases and alcoholism were excluded from the study.

The study was approved by the Ethics Committee of the Michaelidion Cardiac Center, University of Ioannina, Greece and informed consent was obtained from all patients. The study complies with the Declaration of Helsinki.

### Risk factor assessment

All participants underwent a medical interview concerning disease and risk factor history and general use of medications. A physical examination was then performed including measurement of blood pressure, height and weight. Office blood pressure (BP) was measured in the sitting position after 5 min of rest (just before the assessment of arterial stiffness indices) using an automated brachial sphygmomanometer (Omron M7, Omron Healthcare Co, Kyoto, Japan), and the mean of three consecutive measurements by a trained operator was reported. Body mass index (BMI) was calculated as weight/height^2^ (kg/m^2^). The following definitions were used: hypertension; blood pressure (BP) >140/90 mmHg or use of any antihypertensive medications, hypercholesterolemia; low density lipoprotein cholesterol (LDL-c) >2.6 mmol/l (100 mg/dl) or use of lipid-lowering agents (statins). Patients who were smoking at the time of study or had stopped smoking during the last 12 months prior to the study were defined as current smokers.

### Laboratory investigations

Serum fasting glucose was determined by the hexokinase method and HbA1_C_ by a latex agglutination inhibition assay (Randox, UK). Serum total and high density lipoprotein (HDL-c) cholesterol and triglycerides were measured by an enzymatic colorimetric assay. All assays were performed using an Olympus 640 analyser (Olympus Diagnostica GmbH, Hamburg, Germany). LDL-c was calculated using the Friedewald formula: LDL-c = total cholesterol - HDL-c - (triglycerides/5). Serum and urine creatinine concentrations were measured using an enzymatic method. Urinary albumin was measured by a turbidimetric assay. Subsequently, urinary albumin excretion was evaluated by assessment of urine albumin-to-creatinine ratio in a spot sample. Microalbuminuria was defined as urine albumin-to-creatinine ratio between 3.5 – 35 mg/mmol creatinine for at least 2 out of 3 consecutive measurements. The glomerular filtration rate (GFR) was calculated using the chronic kidney disease epidemiology collaboration (CKD-EPI) formula (ml/min/1.73 m^2^) [25]. Serum high sensitivity C-reactive protein (hs-CRP) was measured using rate turbidimetry (IMMAGE Immunochemistry Systems and Calibrator 5 Plus, Beckman Coulter Inc, Fullerton, CA, USA).

### Vascular measurements

All vascular studies were performed early in the morning with the subjects fasted and refrained from smoking for at least 14 h before the study. All measurements were taken in the supine position in a quiet, temperature controlled room (~22°C) after a 30-min period of rest. All studies were performed by the same operator who was unaware of the patient’s medical history.

#### Assessment of endothelial and smooth muscle cell function in the brachial artery

Endothelial function was assessed by measurement of endothelium-dependent flow-mediated dilation (FMD) in the right brachial artery in response to hand hyperaemia. FMD was measured as previously described [26] according to published guidelines [27]. Images of the brachial artery were acquired at baseline, during hand hyperaemia at 90 sec after deflation of a wrist cuff inflated to 300 mmHg for 5 min for measurement of FMD, and at 4 min after 400 μg of sublingual glyceryl trinitrate for measurement of endothelium-independent, nitrate-mediated vasodilatation (NMD). FMD and NMD were calculated as the percent increase in diameter during hyperaemia and after nitrate administration respectively compared with the diameter at rest. Brachial artery blood flow was measured by continuous wave Doppler at rest and at 15 seconds after cuff release. An Echo-Doppler ultrasound system (Ultrasound ATL, HDI 5000, Bophell, WA, USA) and a 5–12 MHz transducer were used for optimal imaging of the brachial artery. Images were recorded on super-VHS videotape (VCR Panasonic AG-MD 835, Osaka, Japan) for off-line analysis. Measurement of brachial artery diameter was performed by another blinded operator, at end-diastole coincident with the R-wave on the electrocardiogram, using electronic calipers from the anterior to the posterior m-line at a fixed distance from an anatomic marker. Internal repeatability data of FMD and NMD measurements in our laboratory have been previously published [26].

#### Assessment of arterial stiffness

Arterial stiffness was assessed non-invasively with the commercially available Sphygmocor system (Version 7.01, At Cor Medical, Sydney, Australia) using applanation tonometry to measure carotid-femoral pulse wave velocity (PWV) as previously described [28]. Pressure waveforms were recorded from the carotid and femoral arteries. The distance travelled by the pulse wave was measured over the body surface as the distance between the two recording sites, and the distance from the suprasternal notch to the carotid was subtracted. Wave transit time (t) between the recording sites was calculated by the system software, using the R wave on the simultaneously recorded electrocardiogram as reference frame. PWV was calculated as distance/transit time. In studies performed on two separate days (8–12 days apart) in 12 subjects by a single operator, the within-subject coefficient of variation of PWV was 5.6%.

### Statistical analysis

Continuous variables are presented as mean ± SD and categorical variables are shown as number (percentage, %). Kolmogorov–Smirnov *Z*-test was used to determine the normal distribution of continuous variables; age, diabetes duration and hs-CRP were not normally distributed. For not normally distributed variables data are presented as median (range). Univariate associations between FMD, NMD, PWV and other studied continuous variables were assessed using Pearson’s and Spearman’s correlation coefficients. For the categorical variables, differences in vascular indices were tested using the unpaired *t*-test. Subsequently, variables whose association with the vascular parameters achieved statistical significance (i.e. *P* < 0.05) as well as other factors that may affect endothelial dysfunction and arterial stiffness (age, gender, heart rate, systolic blood pressure and use of antihypertensive medications) were entered into a stepwise linear regression model to determine independent predictors of vascular parameters. Variables not normally distributed were logarithmically transformed in order to be used in univariate and multivariate association analysis. A *P* value of <0.05 was considered significant. The observed power for the multiple regression analysis given the observed probability level (<0.05), the number of predictors, the observed R^2^ and the sample size, was approximately 85% for FMD and 99% for both NMD and PWV. The SPSS statistical software package (version 15.0 for Windows, SPSS Inc. Chicago, IL, USA) was used.

## Results

Characteristics of subjects with T2DM are presented in Table 1. Median age of our population was 66 years and median time since the diagnosis of diabetes was 10 years. Most of our patients were females (70%), hypertensives (79%) and hypercholesterolemic (91%) with suboptimal control of systolic blood pressure (146±15 mmHg i.e. 68% had systolic blood pressure >140 mmHg and only 5% had diastolic blood pressure >90 mmHg) and LDL-c (3.4±0.9 mmol/L, i.e. 82% had an LDL-c >2.6 mmol/L), while 46% of the total population were obese (BMI >30 kg/m^2^). Good glycaemic control (HbA1c <7%) was observed in 10% of our patients.

**Table 1:** Characteristics of the studied population (n = 165)

***Demographics***	
Age, years	66 (40, 80)
Female gender, n (%)	116 (70)
Time since diagnosis of diabetes, years	10 (1, 33)
Smoking, n (%)	28 (17)
Hypertension, n (%)	131 (79)
Antihypertensive medications, n (%)	109 (66)
ACE-I/ARBs	84 (51)
Calcium channel blockers	58 (35)
Diuretics	24 (15)
Beta blockers	13 (8)
Hypercholesterolemia, n (%)	150 (91)
Statins, n (%)	48 (29)
Oral antidiabetic medication, n (%)	129 (78)
Sulfonylureas	98 (59)
Metformin	89 (54)
Insulin treatment, n (%)	36 (22)
Body mass index, kg/m^2^	30.1 ± 5.5
***Laboratory investigations***	
Fasting glucose, mmoL/L	9.4 ± 2.7
HbA1c, %	8.3 ± 1.2
Total cholesterol, mmoL/L	5.6 ± 1.0
HDL-c, mmoL/L	1.3 ± 0.3
Triglycerides, mmoL/L	1.7 ± 0.8
LDL-c, mmoL/L	3.4 ± 0.9
Creatinine, μmoL/L	78.7 ± 17.7
Glomerular filtration rate, ml/min/1.73 m^2^	78.1 ± 16.3
High sensitivity C-reactive protein, mg/l	1.84 (0.31, 9.71)
Albumin to Creatinine ratio, mg/mmol	1.20 (0.18, 34.50)
Microalbuminuria, n (%)	31 (19)
***Vascular measurements***	
Heart rate, beats/min	72 ± 11
Systolic blood pressure, mmHg	146 ± 15
Diastolic blood pressure, mmHg	78 ± 7
Flow-mediated dilation, %	1.98 ± 1.66
Nitrate-mediated dilation, %	10.09 ± 3.84
Carotid-femoral pulse wave velocity, m/sec	10.2 ± 2.2

Continuous data are presented as mean ± SD. For not normally distributed variables (age, time since diagnosis of diabetes, high sensitivity C-reactive protein and urine albumin to creatinine ratio) data are presented as median (range). *ACE-I*, angiotensin converting enzyme inhibitors; *ARBs*, angiotensin receptor blockers; *HbA1c*, glycated hemoglobin; *HDL-c*, high density lipoprotein cholesterol; *LDL-c*, low density lipoprotein cholesterol.

In our population, FMD was positively associated with NMD (r 0.391, P < 0.001), while PWV was inversely associated with FMD (r −0.218, P = 0.014) and NMD (r −0.309, P < 0.001).

The associations between the vascular indices and other studied parameters are shown in Table 2 (continuous variables) and Table 3 (categorical variables). In multivariate analysis (Table 4), increased duration of diabetes was found to be the single independent predictor of decreased FMD (R^2^ 0.05, P = 0.003), while increased age and fasting glucose as well as the presence of hypertension were independent predictors of decreased NMD (R^2^ 0.16, P < 0.001). Increased age and SBP were independently associated with increased PWV (R^2^ 0.25, P < 0.001) (Table 4).

**Table 2:** Associations between vascular measurements (FMD, NMD and PWV) and other continuous parameters in univariate analysis

	**FMD, %**		**NMD, %**		**PWV, m/sec**	
	**r**	**P**	**r**	**P**	**r**	**P**
*Age, years	−0.155	**0.047**	−0.319	**<0.001**	0.465	**<0.001**
*Time since diagnosis of diabetes, years	−0.254	**0.001**	−0.272	**<0.001**	0.254	**0.004**
Body mass index, kg/m^2^	0.102	0.194	0.027	0.732	−0.217	**0.015**
Fasting glucose, mmoL/L	−0.124	0.113	−0.196	**0.011**	0.024	0.793
HbA1c, %	−0.117	0.135	−0.140	0.072	0.150	0.094
Total cholesterol, mmoL/L	−0.058	0.459	0.082	0.297	−0.131	0.142
HDL-c, mmoL/L	−0.101	0.197	−0.005	0.952	−0.053	0.555
Triglycerides, mmoL/L	0.028	0.724	0.066	0.397	0.034	0.702
LDL-c, mmoL/L	−0.041	0.597	0.068	0.386	−0.143	0.110
Glomerular filtration rate, ml/min/1.73 m^2^	0.073	0.351	0.209	**0.007**	−0.274	**0.002**
*Albumin to Creatinine ratio, mg/mmol	−0.052	0.607	0.025	0.808	−0.013	0.907
*High sensitivity C-reactive protein, mg/l	0.021	0.807	−0.121	0.161	0.057	0.556
Heart rate, beats/min	−0.014	0.856	−0.067	0.393	0.089	0.323
Systolic blood pressure, mmHg	−0.121	0.122	−0.231	**0.003**	0.305	**0.001**
Diastolic blood pressure, mmHg	0.022	0.777	−0.011	0.889	0.014	0.879

*Natural logarithm transformed variables. *FMD*, flow-mediated dilation of the brachial artery; *HbA1c*, glycated hemoglobin; *HDL-c*, high density lipoprotein cholesterol; *LDL-c*, low density lipoprotein cholesterol; *NMD*, nitrate-mediated dilation of the brachial artery; *PWV*, carotid-femoral pulse wave velocity.

**Table 3:** Associations of vascular measurements (FMD, NMD and PWV) with categorical parameters

	**FMD, %**	**P**	**NMD, %**	**P**	**PWV, m/sec**	**P**
Gender						
Male, n = 49	1.92 ± 1.56		10.32 ± 4.52		10.62 ± 2.54	
Female, n = 116	2.00 ± 1.71	0.772	9.99 ± 3.51	0.648	10.02 ± 2.09	0.181
Smoking						
Yes, n = 28	2.01 ± 1.47		10.33 ± 4.07		10.31 ± 2.58	
No, n = 137	1.97 ± 1.70	0.900	10.04 ± 3.80	0.720	10.16 ± 2.18	0.793
Hypertension						
Yes, n = 131	1.93 ± 1.65		9.58 ± 3.58		10.47 ± 2.24	
No, n = 34	2.14 ± 1.73	0.528	12.05 ± 4.19	**0.001**	9.25 ± 1.98	**0.009**
Antihypertensive medications						
Yes, n = 109	1.84 ± 1.64		9.09 ± 3.33		10.54 ± 2.12	
No, n = 56	2.24 ± 1.68	0.143	12.03 ± 4.05	**<0.001**	9.57 ± 2.31	**0.019**
Hypercholesterolemia						
Yes, n = 150	1.94 ± 1.65		10.03 ± 3.86		10.19 ± 2.24	
No, n = 15	2.29 ± 1.77	0.441	10.63 ± 3.66	0.565	10.11 ± 2.23	0.911
Statins						
Yes, n = 48	1.99 ± 1.65		9.88 ± 3.91		10.57 ± 2.45	
No, n = 117	1.97 ± 1.67	0.951	10.17 ± 3.82	0.656	10.01 ± 2.12	0.199
Insulin treatment						
Yes, n = 36	1.53 ± 1.66		8.65 ± 3.01		11.12 ± 2.72	
No, n = 129	2.10 ± 1.65	0.066	10.49 ± 3.95	**0.004**	9.97 ± 2.06	0.059

*FMD*, flow-mediated dilation of the brachial artery; *NMD*, nitrate-mediated dilation of the brachial artery; *PWV*, carotid-femoral pulse wave velocity.

**Table 4:** Determinants of vascular measurements (FMD, NMD and PWV) in multivariate analysis

		**Multivariate analysis**	
		**B (95% CI)**	**P**
FMD, %	Time since diagnosis of diabetes, years*	−0.40 (−0.66, -0.14)	0.003
NMD, %	Age, years*	−5.66 (−9.49, -1.82)	0.004
	Hypertension	−1.80 (−3.25, -0.35)	0.015
	Fasting glucose, μmol/L	−0.36 (−0.54, -0.18)	0.005
PWV, m/sec	Age, years*	6.22 (3.81, 8.63)	<0.001
	Systolic blood pressure, mmHg	0.03 (0.00, 0.05)	0.032

*Natural logarithm transformed variables. *FMD*, flow-mediated dilation of the brachial artery; *NMD*, nitrate-mediated dilation of the brachial artery; *PWV*, carotid-femoral pulse wave velocity.

The independent variables included in the stepwise model were parameters whose association with the vascular parameters achieved statistical significance (*P* < 0.05) and other factors that may affect endothelial dysfunction and arterial stiffness (age, gender, heart rate, systolic blood pressure and use of antihypertensive medications).

## Discussion

In the current study in T2DM patients, indices of vascular function were found to be inter-related suggesting that they probably reflect overlapping pathophysiological aspects of the vascular atherosclerotic damage in T2DM patients. Further to this finding, markers of endothelial function (FMD), smooth muscle cell function (NMD) and large artery stiffness (PWV) were shown to share common correlates. Older age, longer duration of diabetes and treatment with insulin were associated with all markers of vascular dysfunction, although each marker appeared to be independently associated with specific distinct parameters.

Previous studies comparing patients with T2DM to healthy controls have shown that T2DM is an independent risk factor for endothelial dysfunction [14,16,17]. The greater cardiovascular mortality risk observed in T2DM patients has been mainly attributed to vascular endothelial dysfunction [17]. In T2DM patients without macrovascular or microvascular disease, we found that endothelial dysfunction, as assessed by reduced brachial artery FMD, was independently associated only with the duration of diabetes; for every 10 years of diabetes, FMD is reduced by approximately 1.0% (in absolute FMD values). Previous studies have shown significant associations of FMD with diabetes duration [29,30] although not consistently [31]. Longer T2DM duration has also been shown to increase CVD risk and total mortality significantly even after adjustment for established and novel cardiovascular risk factors [32] suggesting that there might be a link between T2DM duration, worsening endothelial function and cardiovascular prognosis in these patients. Furthermore, we showed that impairment of endothelial function in T2DM patients was not related to the presence or levels of various established cardiovascular risk factors, vascular inflammation or glycemic control. In accordance with our findings previously reported data have not shown an association of glycemic control with endothelial dysfunction [31,33]. On the other hand, endothelial dysfunction has been previously related to the presence and levels of cardiovascular risk factors in T2DM patients [23,29,30], although not consistently [34], while vascular inflammation has been suggested to play a central role in the development of endothelial dysfunction [35]. It should be noted that compared to other reports [23,29,30], our study included T2DM patients with severely impaired FMD (mean FMD 1.98%) and a high prevalence of accumulated cardiovascular risk factors; in these patients longer duration of diabetes, and not other established cardiovascular risk factors, was the only important determinant of endothelial dysfunction. Diabetes duration may appear to be a more important contributor to endothelial dysfunction compared to indices of short-term glycaemic control or other isolated risk factors as it may reflect the total exposure of the endothelium to diabetes and hyperglycaemia (metabolic memory) [36] as well as other diabetes-related comorbidities (hypertension, dyslipidemia, obesity).

Vascular smooth muscle cell dysfunction has been previously shown in T2DM patients compared to healthy controls in addition to endothelial dysfunction [11,13,15,37] indicating that T2DM may both reduce the bioavailability of endothelial nitric oxide and attenuate smooth muscle cells’ sensitivity to nitric oxide; these findings have not been consistently replicated in all studies [12,16,18,23]. Vascular smooth muscle cell dysfunction has been previously reported in populations at high cardiovascular risk [38,39] and a prognostic role has been suggested in such individuals [4,10]. In our study, decreased NMD was independently associated with older age, the presence of hypertension and higher fasting glucose. Several previous studies have not shown any significant associations of NMD with risk factors, glycemic control, inflammation or other diabetes-related factors in T2DM patients [13,31]. In agreement with our findings, hyperglycemia was recently reported to be an independent predictor of impaired NMD in T1DM patients [40]. Hyperglycemia, by increasing advanced glycation end-products and oxidative stress in the vascular wall, may be associated with a decreased response of vascular smooth muscle cells [15,35].

Arterial stiffness, as assessed by PWV, has been found to be increased both in prediabetes [41] and in T2DM patients compared to healthy controls [7,20-22]. Increased PWV has been associated with a worse cardiovascular prognosis in high risk patients [3,5,6] including T2DM patients [7,9]. In the present study, increased PWV in T2DM patients was independently associated with older age and higher systolic blood pressure values confirming well established knowledge. Both age and blood pressure are considered to be the two most important determinants of PWV in the general population [42] as well as in T2DM patients [9,21]. In contrast to previous studies we found no association of PWV with glycemic control in T2DM patients [9,21,43,44]. Furthermore, although inflammation, as assessed by hs-CRP, has been suggested to related to increased PWV in healthy subjects [45] as well as in hypertensive [46] and T2DM [47] patients, this has not been replicated in our study.

Our population consisted of patients with T2DM with moderate glycaemic control and less than optimal control of other cardiovascular risk factors (blood pressure, cholesterol, obesity). These results cannot be extrapolated to all patients with T2DM; it is possible that including patients with optimal control of risk factors, important associations of risk factors with vascular indices may be revealed. Finally, whether interventions to improve these risk factors may have a beneficial effect on vascular indices has not been clarified in recent studies [34,48,49], and cannot be precluded from this study.

### Study limitations

This was an observational study that could not reveal causal relationships. Furthermore, regression models in our study could predict a small part of the variability of vascular indices (5–25%) in our population indicating that other factors, not currently studied (e.g. insulin resistance, advanced glycation end-products, genetic factors) may play a more important role in vascular dysfunction in T2DM. Common insulin resistance/sensitivity indices were not assessed because a high proportion of the studied patients were receiving exogenous insulin.

## Conclusion

In conclusion, in T2DM patients without known macrovascular or microvascular disease, impairment of endothelium-dependent vasodilation was independently associated only with longer diabetes duration while no association with other established risk factors known to be related to endothelial function was found. On the other hand, vascular smooth cell dysfunction and increased arterial stiffness were more prominent in older T2DM patients with hypertension. Worse glycemic control was only associated with impaired vascular smooth muscle cell function. Further studies are needed to investigate the clinical and prognostic implications of our findings.

## Abbreviations

T2DM: Type 2 diabetes mellitus; CVD: Cardiovascular diseases; FMD: Flow-mediated dilation; PWV: Pulse wave velocity; NMD: Nitrate-mediated dilation; BMI: Body mass index; BP: Blood pressure; LDL-c: Low density lipoprotein cholesterol; HDL-c: High density lipoprotein cholesterol; GFR: Glomerular filtration rate; hs-CRP: High sensitivity C- reactive protein.

## Competing interests

The authors declare that they have no competing interest.

## Authors’ contributions

KKN participated in the overall design, statistical analysis, data interpretation, writing and presentation of this work. KP participated in the overall design and conduct of the study, data collection and drafted portions of the manuscript. AB performed statistical analysis and contributed to the interpretation of data, writing and presentation of this work. NK participated in data collection, data collection and drafted portions of the manuscript. KP participated in the design of the study, data interpretation and critically revised the manuscript before final approval. ST participated in data interpretation and critically revised the manuscript before final approval. DM participated in study design, data collection and data interpretation. AT participated in study design, data collection, data interpretation and critically revised the manuscript before final approval. LKM supervised the design and conduction of the study, participated in data interpretation and critically revised the manuscript before final approval. All authors read and approved the final manuscript.
